# Platinum drugs and taxanes: can we overcome resistance?

**DOI:** 10.1038/s41420-021-00554-5

**Published:** 2021-06-26

**Authors:** Elena V. Sazonova, Gelina S. Kopeina, Evgeny N. Imyanitov, Boris Zhivotovsky

**Affiliations:** 1 Faculty of Medicine, MV Lomonosov Moscow State University, Moscow, 119991 Russia; 2 Department of Tumor Growth Biology, N.N. Petrov Institute of Oncology, St.-Petersburg, 197758 Russia; 3 Department of Medical Genetics, St.-Petersburg Pediatric Medical University, St.-Petersburg, 194100 Russia; 4 Department of Oncology, I.I. Mechnikov North-Western Medical University, St.-Petersburg, 195067 Russia; 5 Division of Toxicology, Institute of Environmental Medicine, Karolinska Institute, Box 210, 17177 Stockholm, Sweden

**Keywords:** Oncogenes, Cell death

## Abstract

Cancer therapy is aimed at the elimination of tumor cells and acts via the cessation of cell proliferation and induction of cell death. Many research publications discussing the mechanisms of anticancer drugs use the terms “cell death” and “apoptosis” interchangeably, given that apoptotic pathways are the most common components of the action of targeted and cytotoxic compounds. However, there is sound evidence suggesting that other mechanisms of drug-induced cell death, such as necroptosis, ferroptosis, autophagy, etc. may significantly contribute to the fate of cancer cells. Molecular cross-talks between apoptotic and nonapoptotic death pathways underlie the successes and the failures of therapeutic interventions. Here we discuss the nuances of the antitumor action of two groups of the widely used anticancer drugs, i.e., platinum salts and taxane derivatives. The available data suggest that intelligent interference with the choice of cell death pathways may open novel opportunities for cancer treatment.

## Facts

1. Platinum drugs induce at least 5 different cell death modes: apoptosis, necroptosis, mPTP-driven necrosis, ferroptosis, and autophagy.
2. p53, p73, and c-Abl play a key role in cisplatin-induced apoptosis.
3. Taxanes arrest the cell cycle and stop proliferation, inducing cell death via p53.
4. Taxanes could induce autophagy and senescence that decrease therapeutic effects.

## Open Questions

1. How do platinum-containing drugs and taxanes induce nonapoptotic modes of cell death?
2. Can nonapoptotic cell death triggered by platinum-containing drugs and taxanes compensate tumor cell resistance to apoptosis?
3. What are the main capabilities to overcome resistance of tumor cells to taxanes and platinum-containing drugs?

## Introduction

The hallmarks of cancer, which were defined in seminal reviews authored by Douglas Hanahan and Robert Weinberg, include self-induced continuous proliferation, insensitivity to growth suppressors, resistance to cell death, unlimited replicative potential, angiogenesis, invasion and metastasis, genomic instability, inflammation, deregulated cell metabolism and peritumoral immune suppression [1, 2]. Successful targeting of these alterations underlies the development of anticancer treatments. Out of all the hallmarks described above only one, i.e., resistance to cell death, is directly associated with the process of the elimination of cancer cells. The avoidance of apoptosis is the most studied mechanism of cell death escape, although there is growing evidence that some other cell death variants (e.g., necroptosis, ferroptosis, autophagy) may also contribute to tumor cell turnover. The resistance to apoptosis is attributed to dysregulation of Bcl-2 family members (both pro- and antiapoptotic), p53 mutations and upregulation of survival signaling.

Induction of cell death is the key mechanism of the tumor response to targeted therapy, cytotoxic drugs, inhibitors of immune checkpoint control and irradiation. Many tumors demonstrate upfront resistance to therapeutic agents, which at least in some circumstances is related to the downregulation of cell death pathways. Even cancers that initially respond to therapeutic intervention eventually develop drug resistance, often due to the acquired ability to avoid drug-induced cell killing. It has been shown that the drug-induced mitotic catastrophe, which in normal circumstances serves as a pre-stage of various cell death scenarios, at least in some conditions is associated with accelerated tumor cell proliferation [3]. Therefore, interference with various cell pathways may help to combat cancer. Several research groups have shown that inhibition of autophagy may increase the apoptotic response of tumor cells to anticancer treatment [4]. Recent studies demonstrated the utility of inducers of an iron-dependent form of cell death, ferroptosis, for successful elimination of apoptosis-resistant cancer cells [5]. Thus, activation of cell death pathways, which may serve as an alternative to apoptosis, is a promising avenue for development of new approaches for cancer treatment. Here we are focusing on two groups of anticancer drugs that are commonly used in clinical practice and activate different cell death pathways.

## Platinum-containing compounds

Platinum drugs form the backbone for the treatment of ovarian, lung, gastrointestinal and germ-cell cancers and are often utilized for the management of many other types of malignancies. Three platinum derivatives, such as cisplatin, carboplatin and oxaliplatin, have been approved worldwide. Some other agents, e.g., nedaplatin, loboplatin, heptaplatin and miraplatin, have been licensed in selected countries like China, South Korea and Japan [6]. Most laboratory studies devoted to the mechanism of action of platinum drugs utilized cisplatin, which is apparently more potent but somehow also more toxic compared to other compounds of the same class.

The first platinum compound certified for clinical use was cis-diaminodichloroplatinum cis-Pt(NH3)_2_Cl_2_ (cisplatin). Cisplatin was approved as the first metal-containing antitumor drug in 1978 and is used for the treatment of testicular, ovarian, bladder, cervical, head and neck, and small-cell lung cancers [7]. The cytotoxic action of cisplatin is conditioned by the covalent binding of this molecule to N-7 purine atoms on high affinity DNA, which leads to the cross-link of DNA strands and inhibition of DNA synthesis and replication, inducing arrest of the cell cycle and DNA damage response [8]. Carboplatin has less overall toxicity; it contains [cis-Pt(NH3)_2_] active fragment and a bidentate dicarboxylate ligand, which makes it more stable and more hydrophilic. The mechanism of action of carboplatin is similar to cisplatin and accompanied by the formation of 1,2 adducts with the nitrogenous bases of DNA. Carboplatin has a similar spectrum of antitumor activity and exhibits cross-resistance to cisplatin [9, 10]. Oxaliplatin ({[oxalate(2-)-O, O′][1 R,2R-cyclohexanediamine-N, N’] platinum-(II)}) became the third platinum-based anticancer drug. The advantage of oxaliplatin is that it has a different spectrum of action. It is particularly effective against colorectal cancers, which are resistant to cisplatin and carboplatin [11]. Oxaliplatin interacts with DNA by a mechanism similar to cisplatin action. However, the presence of a cyclohexane ligand promotes the formation of adducts, the structure of which is different from that of cisplatin [12].

## Mechanisms of platinum-containing drug action

The main mechanism of action of platinum salts is covalent binding to DNA, which leads to the formation of DNA cross-links and, consequently, inhibition of DNA replication, the cell cycle arrest and cessation of cancer cell proliferation. There were several attempts to improve the antiproliferative effect of cisplatin using its combination with other drugs. It was shown that cardamonin, a chalconoid used for anticancer treatment due to its ability to inhibit mTOR and other prosurvival factors, enhanced the antiproliferative effect of cisplatin [13–15]. In another study, the combination of cisplatin with metformin, a diabetic drug which decreases blood glucose level, appears to inhibit tumor cell proliferation. The molecular mechanisms of this process require further investigations [16].

## Mechanisms of apoptotic cell death induced by platinum-containing drugs

Cisplatin-induced DNA damage is recognized by special cellular machinery, which includes MRE11-RAD50-NBS1 (MRN) complex [17, 18], hMSH2 of the mismatch repair (MMR) complex, the nonhistone chromosomal high-mobility groups 1 and 2 proteins (HMG1 and HMG 2) and the transcriptional factor “TATA-binding protein” (TBP) [19]. The last is a key component of the transcription factor IID, required for the initiation of transcription of all three eukaryotic RNA polymerases [20]. These DNA-damage recognition proteins have been shown to deliver signals from DNA breaks to downstream proteins such as p53, p73 and mitogen-activated protein kinases (MAPK), which activate apoptosis and other forms of cell death [21]. In theory, there are two cellular pathways combating DNA damage, i.e., DNA repair and cell death. Platinum therapy is assumed to deliver extensive DNA damage, which is too severe to be repaired and, therefore, results in cell killing. p53, p73, and MAPK play a central role in converting DNA damage to cell death.

**Fig. 1:**
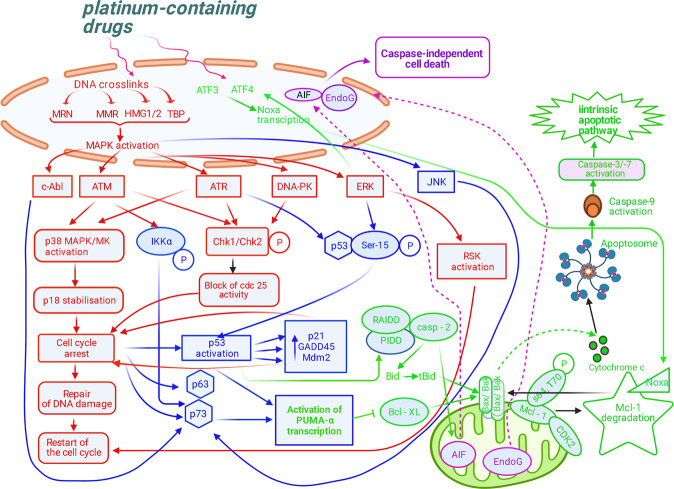
Signal transduction cascades mediating cisplatin-induced apoptosis (for details see in the text). Three kinases, ataxia telangiectasia-mutated protein (ATM), Rad3-related protein (ATR), and DNA-dependent protein kinase (DNA-PK), participate in regulation of the response to DNA damage. Active ATM, ATR, and DNA-PK phosphorylate checkpoint kinases Chk1 and/or Chk2, which block activity of Cdc25, a positive regulator of the cell cycle progression. ATM and ATR are able to activate the p38MAPK/MK2 complex, causing the cell cycle arrest [22]. If DNA damage is repaired, the cell has a chance to restart the cell cycle. If DNA breaks are extensive, the mechanisms of a protection from mutation accumulation and malignant transformation are triggered. Cisplatin-triggered activation of ERKs promotes phosphorylation of p53, which results in overexpression of p21, GADD45, and Mdm2 proteins [27], launching the cell cycle arrest and repair of DNA damage. ERKs also mediate activation of RSK, 90 kDa ribosomal S6 kinase, which promotes proliferation, survival and metastasis [28]. Cisplatin induces stabilization of p18 (Hamlet), a substrate of p38 kinase. These events induce the cell cycle arrest and p53-dependent activation of PUMA-α and NOXA [29–31]. In addition, cisplatin-induced c-Abl upregulation is controlled by MMR proteins and induces p73-dependent activation of PUMA-α transcription. PUMA-α localizes in mitochondria, interacts with and antagonizes an antiapoptotic protein Bcl-XL [32]. Blockage of Bcl-XL function by PUMA-α gives way to Bax and Bak to change their conformation and form pores in the outer mitochondrial membrane, leading to the cytochrome *c* release into cytosol, apoptosome formation, subsequent caspase activation, and subsequent apoptosis [33, 34]. NOXA can be induced by cisplatin treatment in a p53-dependent and independent manner – the last is provided by transcription factors ATF3 and ATF4 [35]. Moreover, cisplatin-mediated caspase-2 activation could lead to the release of apoptosis-inducing factor (AIF) and Endo G from mitochondria. AIF, then translocates to the nucleus to induce cell death in a caspase-independent way [124–126]. Dashed arrows demonstrate translocation. Different apoptotic pathways are highlighted in different colors: DNA damage response pathway is marked in red; p53/p63/p73-dependent apoptosis is marked in blue; intrinsic apoptotic pathway—in green; caspase-independent apoptosis—in purple.

Three kinases, ataxia telangiectasia-mutated protein (ATM), Rad3-related protein (ATR), and DNA-dependent protein kinase (DNA-PK), participate in regulation of the response to DNA damage. Briefly, active ATM, ATR, and DNA-PK phosphorylate checkpoint kinases Chk1 and/or Chk2, which block activity of Cdc25, a positive regulator of the cell cycle progression. Moreover, ATM and ATR are able to activate the p38MAPK/MK2 complex, causing the cell cycle arrest [22]. Then, if DNA damage is repaired, the cell has a chance to restart the cell cycle. If DNA breaks are extensive, the mechanisms of a protection from mutation accumulation and malignant transformation are triggered. In this case, p53 and/or other transcription factors from the same family, such as p63 and p73, are activated and promote expression of genes involved in apoptotic and nonapoptotic cell death [23]. Interestingly, cisplatin treatment preferentially activates the ATR kinase, which phosphorylates p53 on serine-15, resulting in its activation [24, 25]. MAPK signals, including extracellular signal-related kinases (ERKs), c-Jun N-terminal kinases (JNKs), and the p38 kinases, are also involved in cisplatin-induced toxic effects. There are many controversial data about their role in cisplatin-triggered apoptosis [26] which are presented on Fig. 1. On the one hand, the activation of ERKs induces phosphorylation of p53, which results in overexpression of p21, GADD45 and Mdm2 proteins [27], launching the cell cycle arrest and repair of DNA damage. ERKs also mediate activation of RSK, a 90 kDa ribosomal S6 kinase, which promotes proliferation, survival and metastasis [28]. It has also been shown that cisplatin induces stabilization of p18 (Hamlet), a substrate of p38 kinase. This increases the ability of p53 to activate transcription of proapoptotic genes, encoded two BH3-only proteins, such as p53 upregulated modulator of apoptosis (PUMA-α) and NOXA (Latin for damage) [29–31]. PUMA-α localizes in mitochondria, interacts with and antagonizes an antiapoptotic protein Bcl-XL [32]. Blockage of Bcl-XL function by PUMA-α gives way to Bax and Bak to change their conformation and form pores in the outer mitochondrial membrane, leading to the cytochrome *c* release into cytosol, apoptosome formation, subsequent caspase activation, and following apoptosis [33, 34]. It was also shown that Noxa can be induced by cisplatin treatment in a p53-independent manner through transcription factors ATF3 and ATF4 [35].

Another p53-inducible protein, which is activated by cisplatin and has a dual effect on apoptosis, is a p53-induced protein with a death domain (PIDD). This protein interacts with RIP-associated Ich-1/Ced-3 homologous protein with a death domain (RAIDD) and recruits procaspase-2 leading to truncation of Bid by active caspase-2 and following mitochondrial outer membrane permeabilization (MOMP) and apoptosis [36].

p73 also plays an important role in cisplatin-mediated cell death [37]. Because of its structural similarity to p53, it is also considered as a tumor suppressor. Cisplatin-induced accumulation of p73 is dependent on MLH1 MMR proteins since it does not occur upon of their absence [38].

The mechanisms of MMR activation and cell death mediated by p73 upon cisplatin treatment are different from ones realized through p53. Simultaneously, ATM-mediated phosphorylation of nuclear IKK-α, which stabilizes p73, has been suggested to be one of the main apoptotic pathways in response to cisplatin when p53 is lost or mutated [39]. In this case, tyrosine kinase c-Abl can act as a link between DNA damage recognition and p73 activation [40, 41]. Thus, c-Abl is activated by cisplatin and promotes an increase of p73 level; indeed, it was shown that cells with inactive c-Abl are not able to accumulate p73. In addition, cisplatin cytotoxicity is reduced in c-Abl defective cells [33]. The downstream events of both the cisplatin-induced p73 and p53 pathways involve the activation of mitochondria-mediated apoptosis.

## Nonapoptotic forms of cell death induced by platinum-containing drugs

Downregulation of apoptosis induction appears to be the main mechanism of cancer cell resistance to platinum drugs. If DNA-damaged cells cannot undergo apoptosis, the upregulation of alternative cell death pathways may take place. In particular, the disruption of caspase cascade often results in the initiation of necroptosis [42]. Consequently, the support of the necroptotic pathway may help to overcome cancer resistance to therapy. While the initiation of apoptosis requires formation of Death-inducing signaling complex (DISC), apoptosome or p53-inducible death domain containing complex (PIDDosome), necroptosis activation may occur within the Ripoptosome complex, which contains the core components receptor-interacting protein kinase 1 (RIPK1), Fas-associated protein with death domain (FADD), and caspase-8, and assembles in response to genotoxic stress-induced depletion of X-linked inhibitor of apoptosis protein (XIAP), cell inhibitor of apoptosis protein-1/2 (cIAP1 and cIAP2). Ripoptosome assembly requires RIPK1’s kinase activity and can stimulate caspase-8-mediated apoptosis as well as necroptosis if caspases are blocked. DNA damage triggered by the platinum-containing drugs can induce autocrine production of tumor necrosis factor alpha (TNFα), which is usually responsible for Ripoptosome formation. However, this complex could also form upon treatment with Smac-mimetic compounds without involvement of autocrine TNFα. Lobaplatin and cisplatin promote necroptotic cell death via Ripoptosome formation upon inhibition of caspases or cIAP1/2 [43, 44]. RIPK1 is activated within this complex and can activate receptor-interacting protein kinase 3 (RIPK3), which phosphorylates mixed lineage kinase domain-like protein (MLKL). pMLKL monomers oligomerize and form pores in the plasma membrane that lead to cell swelling and disruption [45, 46]. Cisplatin has been shown to induce necroptosis via the formation of necrosome complex only in cell lines that express RIP3 [47]. Deficiency of necroptotic core components—RIPK1, RIPK3, or MLKL—blocks cisplatin-induced cell death as a result of a lack of necrosome formation leading to decreased phosphorylation of MLKL and oligomerization. These data indicate that the treatment with platinum-containing drugs is able to trigger not only apoptosis but directly stimulates RIPK1/3-dependent necrosis. Consequently, the initiation of necrosis may help to overcome tumor resistance to apoptosis upon treatment with this group of chemotherapeutic agents.

**Fig. 2:**
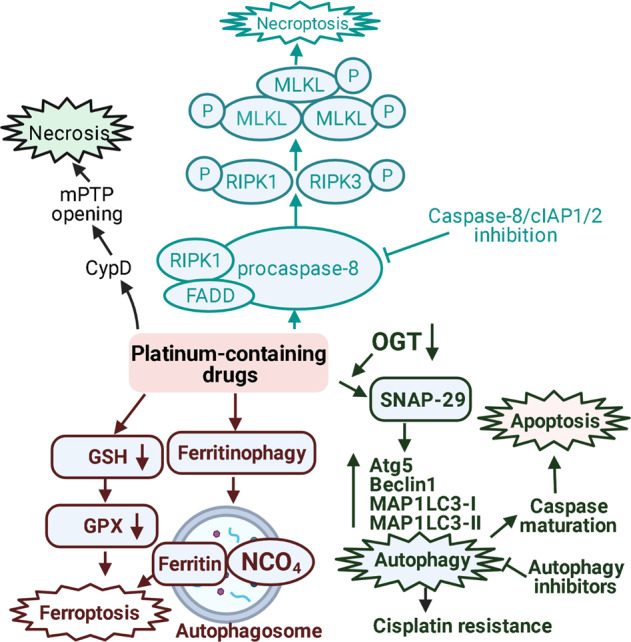
Signal transduction cascades mediating nonapoptotic forms of cell death induced by cisplatin. Cisplatin can stimulate RIPK1/3-dependent necroptosis, mPT‐regulated necrosis, autophagy and ferroptosis. Cisplatin-induced necroptosis activation occurs within Ripoptosome complex (consisting of RIP1, FADD, and procaspase-8) upon inhibition of caspases or cIAP1/2, which promotes necrotic cell death [43, 44]. It is negatively regulated by FLIP, cIAP1, cIAP2, XIAP and caspase-2 [44]. Mechanistically, IAPs target components of this complex for ubiquitylation and inactivation [127, 128]. RIPK1 is activated within this complex and can activate RIPK3 which promotes phosphorylation of MLKL. pMLKL monomers oligomerize and form pores in the plasma membrane that lead to cell swelling and disruption [46, 47.Along with RIPK1/3-dependent necroptosis, cisplatin is able to trigger mPT‐regulated necrosis, which involves the opening of the mPTP and is mediated by cyclophilin D (CypD) [23]. Cisplatin induces autophagy, which is provided by the downregulation of O-linked N-acetylglucosamine transferase (OGT) via SNAP-29, resulting in upregulation of Beclin 1, Atg5 and MAP1LC3-I and increased conversion of MAP1LC3-I to MAP1LC3-II [57]. Autophagy blockage by inhibitors like LY294002 or 3-methyladenine or wortmannin can stimulate caspase activation and apoptosis [61]. Cisplatin can deplete intracellular glutathione that leads to glutathione peroxidases (GPX) inhibition and ferroptosis induction [64]. Cisplatin-induced ferroptosis may develop as a result of ferritinophagy [5]. This process activates the binding of ferritin to NCOA_4_ in the autophagosome and delivers it into the lysosomes, activating the ferroptosis process [65]. Different nonapoptotic forms of cell death induced by cisplatin are highlighted in different colors: necroptosis pathway is marked in turquoise, necrosis is marked in black; ferroptosis—in brown; autophagy—in dark green.

Importantly, cisplatin can induce another form of necrotic cell death—mitochondrial permeability transition (mPT)‐regulated necrosis, which is associated with a rapid increase in mitochondrial membrane permeability and involves the opening of the mPT pore (mPTP) [23]. Deficiency of cyclophilin D (CypD), a key player of mPTP opening, partially reduced cisplatin-induced necrosis, indicating an essential role of CypD mediated-mPTP opening for cancer cell demolition after chemotherapy [48]. mPTP-dependent necrotic death does not involve RIPK1/3 or MLKL directly but can develop together with RIPK1/3-dependent necroptosis due to autocrine production of TNFα [48]. The question of whether CypD- and RIPK1/3-mediated pathways interfere or are triggered individually is still open. On the one hand, in a mice model of ischemia-reperfusion injury, cisplatin has been shown to induce both forms of cell death independently [49]. On the other hand, RIPK3 could be involved in mPTP opening independently on RIPK1 and MLKL [50]. In addition, deficiency of both CypD and TNFα blocked cisplatin-induced cell death, suggesting that this agent could induce necrosis through TNFα-dependent and -independent pathways. These findings provide new insight into the molecular mechanisms underlying the sensitivity of tumors to cisplatin treatment [48]. The detailed mechanism of this process is presented on Fig. 2. Importantly, cancer cells can avoid necrosis. Thus, ubiquitination of RIPK1 by the E3 ligase c-IAP1/2 activates the canonical NF-κB pathway, resulting in cell survival [51]. It may be considered as one of the mechanisms of resistance to cisplatin-mediated cell death.

Platinum-containing agents are able to induce not only apoptotic or necrotic cell death but also stimulate autophagy. Autophagy is a physiologically regulated and evolutionarily conserved process that plays a critical role in the degradation of cellular components, proteins and other macromolecules [52, 53]. This process involves the sequestration of proteins and organelles in double-membraned structures, termed autophagosomes, in the cytoplasm to target them to the lysosomes for degradation in autophagolysosomes. The formation of an autophagosome is mediated by the induction of several autophagy gene products, including MAP1LC3, phosphatidylinositide 3-kinase, Beclin 1, and a network of other Atg (**A**u**T**opha**G**y) genes initially identified in yeast (Fig. 2) [54].

Close association between autophagy and cancer has been described, where it plays an important but contradictory role in cell survival and death. Autophagy is strongly activated by various stress conditions, including cancer chemotherapy and radiotherapy [55]. Drug-induced autophagy may have a protective role, maintaining cell homeostasis and survival, which decrease efficiency of the therapy [56]. Thus, recent studies have reported that downregulation of O-linked N-acetylglucosamine transferase (OGT) enhances cisplatin-induced autophagy, resulting in a development of cisplatin-resistant ovarian cancer [57]. Interestingly, activation of autophagy as well as activation of apoptosis can be realized through MAP kinases such as ERK1/2, p38 and MAPK8/JNK1 (c-Jun N-terminal Kinase) (Fig. 2) [58].

Autophagy can play a dual role in the process of anticancer therapy. On the one hand, the switch of apoptosis to autophagy is a factor of tumor cell survival, which contributes to the formation of resistance to cisplatin therapy. T24 cancer cells derived from patients, whose tumor was resistant to cisplatin, were characterized by the intensification of autophagy [59]. In addition, a dose-dependent increase of autophagy level in human bladder cancer cell lines RT-4 and 5637 was shown in another study [60]. It is reasonable to conclude that cisplatin is able to induce protective autophagy in human cancer cells, and this process may represent a mechanism of resistance to platinum-containing agents. On the other hand, autophagy can protect renal tubular epithelial cells (RTECs) from cisplatin injury to reduce severe therapy-induced side effects, such as nephrotoxicity. Yang et al. examined the expression of autophagic proteins and the formation of autophagosomes upon cisplatin injury of RTECs [61]. An upregulation of Beclin 1, ATG5 and MAP1LC3-I and increased conversion of MAP1LC3-I to MAP1LC3-II were observed at 2–4 h after cisplatin injection, indicating the activation of autophagy (Fig. 2). The appearance of cisplatin-induced punctated staining of autophagosome-associated MAP1LC3-II upon GFP-MAP1LC3 transfection in RTECs that was blocked by the autophagy inhibitor 3-methyladenine, provided further evidence of autophagy flux. Moreover, the formation of numerous acidic autophagolysosomes was observed through considerable red fluorescence in cisplatin-treated cells after staining with acridine orange, normally displaying green fluorescence in the cytoplasm and nucleus. Autophagy inhibitors LY294002 or 3-methyladenine or wortmannin inhibited the formation of autophagosomes but induced apoptosis after 2–4 h of cisplatin treatment, as indicated by caspase-3/−7 and −6 activation and nuclear fragmentation. This switch from autophagy to apoptosis provided by inhibitors suggests that the preapoptotic lag phase after drug administration is mediated by autophagy. In addition, apoptosis was shown to be linked with autophagy at later stages of cisplatin injury since inactivation of Beclin 1 and ATG5 by autophagic inhibitors enhanced caspase maturation and subsequent cell death [61].

Rovetta and co-authors used a multidisciplinary approach to study the correlation between autophagy and apoptosis in normal rat renal cell line NRK-52E exposed to cisplatin. The authors showed that the development of apoptosis or autophagy depends on the amount of cisplatin. So a low drug concentration (10 μM) triggered autophagy while a 5-times higher concentration (50 μM) resulted in the induction of apoptosis. Interestingly, co-administration of cisplatin with 3-methyladenine promoted apoptosis, whereas the preconditioning of 50 μM cisplatin with taurine resulted in apoptosis rescue and a shift to autophagy [62]. Thus, it was shown that autophagy can protect kidney cells from cisplatin injury. The detailed analysis of specific endoplasmic reticulum (ER) markers GRP78, GRP94, and GADD153/CHOP expression revealed a possible role of ER signaling cascade in the cisplatin-induced crossover between apoptosis and autophagy that makes possible the development of new strategies to reduce platinum-containing drug nephrotoxicity [62].

Ferroptosis is a mechanism of programmed cell death induced by chemotherapeutic drugs which was discovered ~10 years ago [63]. It has been shown that cisplatin can deplete intracellular glutathione that leads to glutathione peroxidases (GPX) inhibition and induce ferroptosis [64]. Cisplatin-induced ferroptosis may develop as a result of ferritinophagy, a process of ferritin degradation, which increases the level of free iron [5]. This process in turn induces the binding of the iron storage protein ferritin to NCOA_4_ in the autophagosome and delivers it into the lysosomes, activating the ferroptosis process (Fig. 2) [65]. Ferroptosis inducers such as erastin or RSL3 have been shown to enhance the anticancer effects of cisplatin during the treatment of various cells from lung, colorectal, ovarian, and pancreatic ductal adenocarcinoma cancers through inhibition of system Xc- or GPX4 [66].

More and more evidence has revealed the crosstalk between autophagy and ferroptosis. Most existing studies suggest that autophagy promotes drug resistance, while ferroptosis is generally considered as a mechanism that could restore cancer sensitivity to chemotherapy. Therefore, the question of which strategies are able to tilt the balance between ferroptosis and autophagy toward the first one to combat drug resistance is still open [67].

Taken together, it is possible to conclude that platinum-containing drugs trigger different processes that block tumor progression and promote its demolition. In the first instance these medications stop uncontrolled cell cycle progression by inhibiting proliferation. Since DNA damage induced by the drugs cannot be repaired, the various cell death modalities including not only apoptosis but also autophagy, necroptosis and ferroptosis, are induced. The relationship between these types of cell death can play both a positive and negative role in chemotherapy because apoptosis, necroptosis and ferroptosis destroy tumor cells, while autophagy in some cases can protect them. However, at the same time, autophagy protects not only cancer cells but also normal cells, so it should not be rashly inhibited.

## Taxanes and their different anticancer effects

Taxanes (paclitaxel (PTX), docetaxel and cabazitaxel) are widely used in clinical practice for the treatment of ovarian, breast, lung cancers and many other tumor types [68]. Unlike most antitumor drugs, which damage DNA or RNA, the main mechanism of taxanes’ action is the promotion of cellular death through the binding of tubulin and the inhibition of the microtubules’ disassembly, which are required for chromosome segregation and cell division [69, 70]. Therefore, taxanes belong to the group of the so-called microtubule targeting agents (MTAs) [71, 72]. The microtubules are tubulin heterodimers involved in many important cellular processes. The production of tubulin and the microtubules assembly occurs during the G2 phase of the cell cycle [73]. MTAs can be classified into microtubule stabilizing agents, such as taxanes, and destabilizing agents, such as vinca alkaloids, which bind to the α/β tubulin in order to disassemble microtubules [74]. Although both groups of MTAs induce cell death and are widely used as anticancer agents, they have opposite mechanisms of action [75]. In fact, cells exposed to taxanes are detained in the cell cycle progression, so the main mechanism of their action is the inhibition of proliferation [68, 76]. Below we focus on the mechanisms of different forms of cell death, which are activated after the taxane-induced block of the cell cycle.

The PTX treatment may lead to the cell cycle arrest at different phases depending on the dose used. It is well known that this drug blocks cells in the G2/M phase. However, low doses of PTX are able to detain the cell cycle at G0/G1 in several types of tumor cells (e.g., HT29-4D colon carcinoma cells, HL-60 promyelocytic leukemia cells and ovarian cancer cells), indirectly downregulating expression of c-Myc oncogene [77, 78]. However, it is important to emphasize that cells can carry out a premature mitotic exit known as mitotic slippage, which is the major mechanism of rescue cells from MTAs, limiting the therapeutic efficacy of these drugs [79]. Moreover, after mitotic slippage, cells can gain enhanced malignancy due to acquired mutations that worsen prognosis of the disease.

## Mechanisms of apoptotic cell death induced by taxanes

**Fig. 3:**
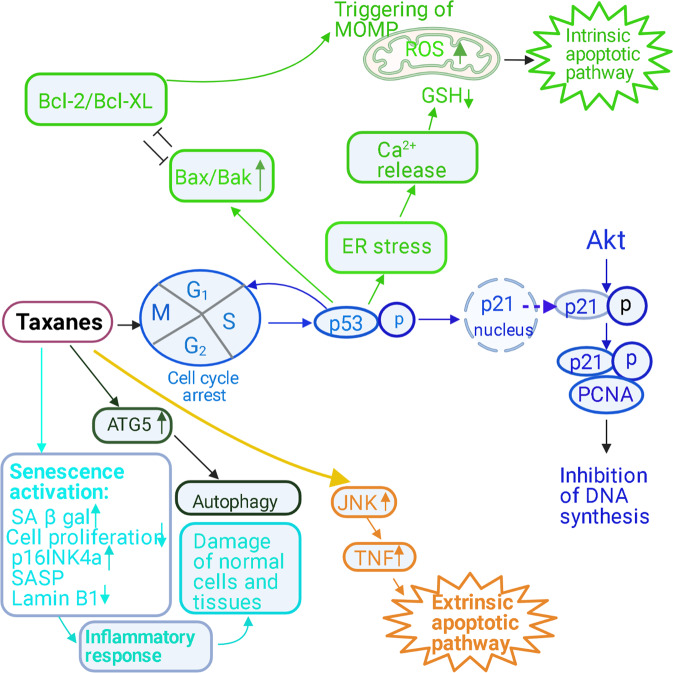
Signal transduction cascades mediating taxane-induced cell death. Taxane treatment blocks cell proliferation, induces apoptosis and different types of cell responses to stress—autophagy, senescence and inflammation. Low-dose PTX treatment induces p53-mediated translocation of p21 into the cytoplasm where it is phosphorylated by Akt [84] and inhibits the cell cycle progression and DNA synthesis via binding PCNA [88]. PTX induces a downregulation of the antiapoptotic protein Bcl-2 and an upregulation of the proapoptotic protein Bax [81, 95]. These alterations are responsible for the triggering of MOMP and the stimulation of the intrinsic apoptotic pathway [96]. In addition, PTX promotes ER stress [93]. Possibly, ER stress derives from gene expression modulation caused by direct paclitaxel-mediated p53 activation. Damage of the ER may cause a release of Ca^2+^ that provokes Ca^2+^ overload and subsequent mitochondrial damage, leading to increased ROS production [94]. PTX stimulates nonlethal cell response to stress like autophagy via upregulation ATG5 [105]. In addition, senescence is induced by PTX manifested in increased senescence-associated beta-galactosidase (SA-βgal) activity, depressed cell proliferation, increased p16 INK4a expression, and senescence-associated secretory phenotype (SASP) factors such as (Il1α, Il6, Mmp3, Mmp9, Cxcl1, Cxcl10, and Ccl20), as well as downregulation of Lamin B1 [119]. The latter is an E2F target gene [129] and a decrease of its expression during senescence is induced by oncogenic Ras and mediated by p53 and a retinoblastoma protein pRb [130]. Dashed arrow demonstrates translocation. Different types of cell death induced by taxanes are highlighted in different colors: intrinsic apoptotic pathway is marked in green; extrinsic apoptotic pathway—in light brown; p53-dependent apoptosis is marked in blue; autophagy—in dark green; senescence—in light blue.

Although mechanisms of how taxanes trigger cell death outside division have been reported, pro-death signals frequently accumulate during extended mitotic arrest upon taxane treatment [80]. It is not entirely clear how these agents induce apoptosis; however, apoptosis has been suggested to be related to p21 activation and induction of the following molecular pathways [81]. It should be noted that PTX possesses very low water solubility [82] and its plasma concentration is nanomolar [83], so in vitro studies of the drug action is relevant for only low doses of this drug. Low doses of PTX were found to inhibit the proliferation of some tumor cell lines, like HCT116 and RPMI 2650 and be accompanied by increased levels of p21 but slow or little the cell cycle perturbation (Fig. 3) [84, 85]. For example, after 3 days of incubation with extremely low-dose PTX (0.1–0.3 nM), essential p21 upregulation was detected but only a small portion of cells accumulated in G1 phase [85]. These controversies can be explained by the fact that upon low-dose PTX treatment p21 translocates into the cytoplasm where it is phosphorylated by Akt [84]. This modification possesses an inhibitory effect on the cell cycle controlling functions of p21 [86, 87]. However, p21 is able to not only regulate the cell cycle progression but inhibit DNA synthesis via binding PCNA [88] that might compensate the decrease of p21’s ability to block the cell cycle (Fig. 3). Importantly, absence of p21 promotes a development of spontaneous tumors in mice [89] and mutations in this gene were shown to correlate with PTX resistance in mammary cells [90]. However, p21 does not always show tumor suppressor functions: its role depends on the cellular context, p21 subcellular localization and post-translational modifications [91]. In addition, the level of p53 as p21 positive regulator was also shown to be significantly upregulated by low doses of PTX [92]. As noted above, p53 acts as a direct trigger of cell death via its different targets (PUMA-α, NOXA, PIDD). Taken together, the doses of PTX relevant for in vivo are able to induce p21- and p53-mediated cell response but detailed mechanisms will require further study.

According to a recent study, PTX promotes accumulation of reactive oxygen species (ROS) and an overexpression of the genes linked to ER stress in osteosarcoma cells [93]. However, it remains unclear whether ER stress derives from gene expression modulation caused by direct paclitaxel-mediated p53 activation. In addition, damage of the ER may cause a release of Ca^2+^ that provokes its overload and subsequent mitochondrial damage, leading to increased ROS production (Fig. 3) [94]. Furthermore, another study has demonstrated that in canine mammary gland tumor cells, PTX induces a downregulation of the antiapoptotic protein Bcl-2 and an upregulation of the proapoptotic protein Bax [81, 95]. These alterations are responsible for the triggering of MOMP and the stimulation of the intrinsic apoptotic pathway [96].

## Nonapoptotic forms of cell death induced by taxanes

The mitochondrial network is crucial for the modulation of the cross-talk between autophagy and apoptosis and can regulate cell death or survival decision [97]. Studies of mitochondrial network redistribution have demonstrated that docetaxel induces mitochondria fission, which mediates several biological phenomena, including apoptosis and autophagy [98, 99]. It was noted above that there is a great controversy about the role of autophagy in the death and survival of tumor cells. Although some researches assure that paclitaxel-induced autophagy is one of the antitumor mechanisms for killing cancer cells, others suggest that it is a pathway for cell survival [100–102]. It has been reported that autophagy contributes to resistance development in response to PTX treatment [103]. Importantly, autophagy modulators are able to improve the therapeutic effect of PTX [104]. Accordingly, the molecular mechanisms underlying autophagy induction in tumor cells during PTX resistance development should be studied more deeply. Thus, the ATG5 has been demonstrated to be involved in the autophagic response induced by PTX (Fig. 3) [105]. *ATG5* gene knockout (KO) in v-Ha-*ras*-transformed NIH 3T3 cells with multidrug resistance (Ras-NIH 3T3/Mdr) restored sensitivity of these cells to PTX. Remarkably, exogenous expression of *ATG5* restored process of autophagy in *ATG5* KO cells but failed to rescue PTX sensitivity. These results indicate that resistance to a low dose of PTX in *ATG5* KO cells may be related to its other functions, which are independent on its role in autophagy. Thus, the *ATG5* KO was shown to promote the G2/M arrest of the cell cycle. In addition, *ATG5* KO underwent necrosis in a high number of cells after PTX treatment. These data suggest that the difference in PTX sensitivity between *ATG5* KO and their parental cells may result from the disparity in the proportions of necrotic cells in both populations. Thus, these results demonstrate that the *ATG5* KO in multidrug-resistant cells leads to a marked G2/M arrest and sensitizes cells to PTX-induced necrosis [106].

As noted above, necroptosis can compensate inhibited apoptosis and be activated in response to many anticancer drugs in cancer cells expressing RIP1 and RIP3 kinases [107, 108]. Taxane treatment is able to promote necroptosis although the mechanism remains poorly understood [109]. Therefore, JNK signaling pathway may mediate the transcription of transmembrane TNF launched by MTAs [110]. TNF-mediated tumor cell killing was shown to be reinforced by IAP antagonists (Smac mimetics) in the majority of cancer types. This process was well studied in patient-derived xenograft (PDX) models, which are most similar to human tumors [110]. It was also shown that in response to docetaxel, MDA-MB-231 cells stably expressing ectopic Bad have extended mitotic arrest that led to cell death in mitosis. At the same time, mitotic slippage of this cells was decreased. It was surprising that this death was nonapoptotic and not dependent on activation of caspases or Bcl-XL interaction. This cell death had necroptotic features and was mediated by ROS. So, inhibition of caspases did not attenuate cell death, which is consistent with necroptotic signaling. Blockage of necroptosis with the MLKL inhibitor necrosulfonamide significantly decreased cell death in Bad-overexpressed cells only, indicating that this protein stimulates docetaxel-induced necroptosis [111]. The addition of both a pan-caspase inhibitor, z-VAD-FMK, and necrosulfonamide significantly reduced cell death in both control and Bad-expressing cells, confirming that one mechanism of cell death might be activated when the other is compromised. These results suggest that Bad expression might be a prognostic factor for favorable outcome in response to taxane chemotherapy by enhancing necroptotic cell death and inhibiting the proliferation of potentially chemoresistant polyploid cells [112].

As mentioned above, PTX could inhibit the proliferation of tumor cells through blocking the cell cycle G0/G1 or G2/M phases. The low-dose treatment induced strong antiangiogenic and anti-lymphangiogenic activities in vitro (human ovarian cancer cell lines, HUVEC) and in vivo (human oral squamous cell carcinoma, 4T1 metastatic breast cancer) instead of mitotic arrest or apoptosis of tumor cells [78, 113]. Furthermore, it was shown that low doses of PTX suppress genes associated with glutaminolysis, inhibiting the growth of tumor cells, and also increases the level of cellular lactate [85, 114, 115].

Taxanes are able to induce not only apoptotic or necrotic cell death but also stimulate senescence, which is characterized by the cessation of cell division. Senescent cells are characterized by several features: resistance to apoptotic stimuli; irreversible arrest of growth; increased activity of lysosomes; metabolism dysregulation; persistent DNA damage; elevated chemokine, growth factor and cytokine secretion [116]. Many chemotherapeutic drugs induce senescence in cancer cells and the tumor microenvironment. Therapy-induced senescence (TIS) can stimulate immunosurveillance to eliminate tumor cells, but can also be a source of chronic inflammation and drug resistance [117]. Indeed, it was shown that PTX treatment of MCF7 breast cancer cells [118] and various nonmalignant cell lines, such as endothelial cells, bone marrow mononuclear cells, and human and mouse fibroblasts, induces senescence (Fig. 3) [119, 120]. Senescence was shown to be induced by PTX in mouse cells, manifested in increased senescence-associated beta-galactosidase (SA-βgal) activity, depressed cell proliferation, increased p16 INK4a expression, and senescence-associated secretory phenotypes (SASP) factors. The proliferation defects and senescence induced by decreased levels of Lamin B1 and microtubule disruption can induce alteration of mitochondrial integrity and ROS production [121]. This in turn may result in the death of tumor cells by apoptosis [122]. On the other hand, it might have a negative consequence, leading to the development of an inflammatory response, accelerating the damage of normal cells and tissues [123]. Moreover, the drugs can induce senescence in normal tissues in vivo that could play a negative role in tumor progression because the SASP pattern in tumor microenvironment stimulates cancer progression [23]. It was also shown that PTX, temozolomide and cisplatin also elevated p16 INK4a expression in skin, which indicates the formation of a senescent status of cells. These data revealed that cytotoxic chemotherapeutic agents with different mechanisms of action, including taxanes and platinum-containing compounds, can trigger senescence of normal and malignant cells in different tissues in vivo that may worsen prognosis of the disease. Thus, the relationship between apoptosis, necrosis and senescence can play both positive and negative roles in chemotherapy, because apoptosis and necroptosis destroy tumor cells, while senescence being a source of chronic inflammation can indiscriminately damage not only tumor cells, but also participate in the formation of their resistance to treatment.

## Concluding remarks

Cancer is a consequence of multiple deregulated processes, first defined as the “Hallmarks of Cancer” by Hanahan and Weinberg two decades ago, implicating six main features, and then amended to ten [1, 2]. There are a large number of medications used to treat both hematological and solid tumors, but their efficacy leaves a great deal to be desired. The key problem of neoplasm chemotherapy is the absence of specific therapeutic targets and the formation of resistance to the ongoing treatments. The apoptosis inhibition seems to be the main cause of drug insensibility. Fortunately, as many studies have shown, the disturbances of this cell death type open a window for triggering other processes, which are able to combat cancer cells. Thus, necrotic death modalities—RIP1/3-mediated necroptosis, mPT‐regulated necrosis and ferroptosis—induced by platinum-containing compounds and taxanes can improve the outcome of the anticancer chemotherapy. Simultaneously, the same medications might promote the development of autophagy and senescence—nonlethal forms of cell response to stress. These processes protect tumor cells from death decreasing the therapeutic effect. Importantly, the mechanisms of how these agents induce a switch between different forms of cell death and response are poorly understood and require in-depth investigation both in vitro and in vivo. However, we are positive to suggest that the moderation of the drug dose and/or administration regime influences pathways of tumor cell killing and may help to overcome drug resistance.
